# Campho-Phenique as a Local Anesthetic

**Published:** 1907-04

**Authors:** B. L. Thorpe


### CAMPHO-PHENIQUE AS A LOCAL ANESTHETIC.

All operations in the mouth should be preceded by the application of a local anesthetic. In many mouths nausea, even vomiting, is caused by the insertion of fingers, instruments, or impression trays.

To avoid this especially is campho-phenique indicated to obtund local sensation and prevent pain incident to the removal of calculus, polishing teeth, wedging, applying and ligating the rubber dam, removing loose roots, and to prevent nausea, gagging, etc., prior to taking impressions.

Nothing to the writers knowledge acts so nicely as ten grains of finely powdered cocain added to one fluid-ounce of campho-phenique (origingal package). This makes approximately a two per cent. solution, which, when applied on a large sized pledget of cotton to the entire mucous surface of the mouth, completely obtunds local sensation and overcomes nausea.

In cases of odontalgia from an exposed pulp a warmed application of this solution on a pellet of cotton usually relieves the pain, and for treating sensative cavities preparatory to filling it is of equal value when the remedy is sealed in the cavity with temporary stopping of gutta-percha.—B. L. Thorpe, Brief.

